# Conserved A-to-I RNA editing with non-conserved recoding expands the candidates of functional editing sites

**DOI:** 10.1080/19336934.2024.2367359

**Published:** 2024-06-18

**Authors:** Yuange Duan, Ling Ma, Tianyou Zhao, Jiyao Liu, Caiqing Zheng, Fan Song, Li Tian, Wanzhi Cai, Hu Li

**Affiliations:** Department of Entomology and MOA Key Lab of Pest Monitoring and Green Management, College of Plant Protection, China Agricultural University, Beijing, China

**Keywords:** A-to-I RNA editing, conserved, recoding, selection, evolution

## Abstract

Adenosine-to-inosine (A-to-I) RNA editing recodes the genome and confers flexibility for the organisms to adapt to the environment. It is believed that RNA recoding sites are well suited for facilitating adaptive evolution by increasing the proteomic diversity in a temporal-spatial manner. The function and essentiality of a few conserved recoding sites are recognized. However, the experimentally discovered functional sites only make up a small corner of the total sites, and there is still the need to expand the repertoire of such functional sites with bioinformatic approaches. In this study, we define a new category of RNA editing sites termed ‘conserved editing with non-conserved recoding’ and systematically identify such sites in *Drosophila* editomes, figuring out their selection pressure and signals of adaptation at inter-species and intra-species levels. Surprisingly, conserved editing sites with non-conserved recoding are not suppressed and are even slightly overrepresented in *Drosophila*. DNA mutations leading to such cases are also favoured during evolution, suggesting that the function of those recoding events in different species might be diverged, specialized, and maintained. Finally, structural prediction suggests that such recoding in potassium channel Shab might increase ion permeability and compensate the effect of low temperature. In conclusion, conserved editing with non-conserved recoding might be functional as well. Our study provides novel aspects in considering the adaptive evolution of RNA editing sites and meanwhile expands the candidates of functional recoding sites for future validation.

## Introduction

### A-to-I RNA editing in metazoans

Adenosine-to-inosine (A-to-I) RNA editing is highly abundant in the mRNAs of metazoans [1–3]. Since I is read as G, A-to-I RNA editing in CDS is able to ‘recode’ the protein sequence, termed recoding sites [4–6]. Particular recoding events can have a strong impact on protein function and the fitness of organisms [7–10]. Although the numbers and distributions of RNA editing sites vary widely across distantly related species, this editing pathway is generally conserved in metazoans. Adenosine deaminase acting on RNA (ADAR) mediates the editing on non-tRNA RNA molecules [11,12]. The ADAR protein family typically contains a deamination domain and several dsRNA-binding domains, enabling the enzyme to recognize dsRNA and catalyse the deamination reaction. The targets of ADARs are mainly neuronal and nervous system-related genes and thus usually the head/brain transcriptomes are prioritized for RNA editing detection [1,2]. Mammals have three ADARs, among which ADAR3 is mammal-specific and has no catalytic activity [13]. In insects, the common ancestor lost ADAR1 and therefore all extant insects only have one *Adar* gene which is orthologous to mammalian ADAR2 [14]. Given the deep conservation of editing pathway within different animal clades, it is not surprising to observe considerable highly conserved RNA editing events that were inherited and maintained from the common ancestor of a particular clade.

### Conserved recoding sites are likely to be functional

Through decades of studies on functional RNA editing sites together with the recent eruption of omics data, researchers found that highly conserved recoding sites in different clades usually show strong signals of functional importance and positive selection. For example, the Q>R recoding in glutamate receptor *GRIA2* gene is strictly required for the survival of mice [15–18], and in all tested mammalian species, the recoding level is nearly 100% in brains [19,20]. The genomically encoded AA (Gln) alone is lethal, and only the fully edited Arg version is acceptable for the organisms [21]. In addition, an I>V recoding site was found to be conserved in cephalopods with ~300 Mya divergence. The relative proportions of Ile and Val isoforms were adjusted by the differential editing levels across different species or populations [7,22]. Functional experiments showed that this I>V recoding site could ‘normalize’ the closing rate of potassium channel Kv2 in different species [23].

These conserved and functional recoding sites leave us an impression that (1) the original AA sequences and post-edited AAs have to be the same across species in order to show the conservation, function, essentiality, and adaptiveness of the recoding site and that (2) the DNA mutations in the edited codon (not necessarily at the editing site) are intuitively to be deleterious because this mutation either abolishes the ability to be edited or changes the original AA to an unrelated/non-functional AA.

### Conserved editing at nonsynonymous sites does not necessarily imply conserved recoding

**Figure 1.**
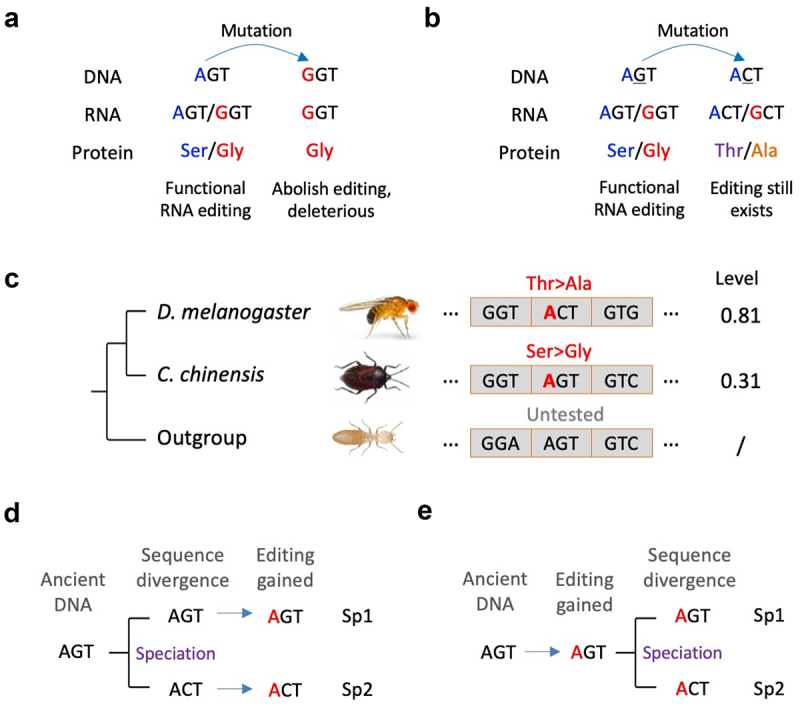
Definition of conserved editing with non-conserved recoding. (a) The DNA mutation on an existing RNA editing site abolishes the editing potential and thus is deleterious. (b) The DNA mutation next to an existing RNA editing site leads to a different codon change by RNA editing. (c) The known case of conserved editing with non-conserved recoding between D. melanogaster (Diptera) and C. chinensis (Hemiptera) [24]. Codon alignment is shown. (d) A less likely situation where conserved editing was independently gained after sequence divergence of two species. (e) A more likely situation where the ancient RNA editing existed before the sequence divergence of two species.

DNA mutations taking place on existing RNA editing sites would directly abolish the editing potential. If this editing site is a conserved recoding site with putative function, then the destructive DNA mutations, which are also nonsynonymous mutations, are likely deleterious (Figure 1(a)). Although very rare, these cases are systematically studies in the phylogeny of cephalopods [25]. However, a less studied case is the nonsynonymous mutations taking place next to the editing site and within the same codon (Figure 1b). For example, consider an A-to-I recoding site at the first codon position, if the original codon is AGT (Ser) and can be edited to GGT (Gly), and a G>C DNA mutation at the 2nd codon position changes AGT to ACT (Thr), but the first codon position still has the editing potential, then the recoding will be from ACT (Thr) to GCT (Ala) (Figure 1b). Given sufficient time for selection, drift, and speciation, one might observe that an A-to-I editing event is conserved between two species, but it recodes different AAs in different species. Indeed, this is what we actually saw in gene *Shab* between *Drosophila melanogaster* and a hemipteran species *Coridius chinensis* (Figure 1c) [24]. Although only one case was found between the two species, we defined this case as ‘conserved editing with non-conserved recoding’. It is unclear whether this type of mutation is deleterious, beneficial, or nearly neutral since we do not know whether the recoding event in the new codon context is functional. Moreover, regarding how the non-conserved recoding emerged in different species, it is unlikely that the DNA sequences of two species first diverged and then RNA editing independently gained at the same position (Figure 1d). Instead, a more plausible evolutionary trajectory is that this recoding event was gained in the common ancestor and then DNA mutation occurred after speciation (Figure 1e). Thus, given the existence of this situation, it should be noted that even if a nonsynonymous editing event is conserved across different species, the ‘type of recoding’ is not necessarily the same because of different sequence context.

### Aims, scopes, and novelties

Regarding the evolutionary pressure acting on conserved editing with non-conserved recoding, question comes that if an anciently conserved recoding event was highly essential due to the delicate functional switch caused by the particular AA changes (e.g. Q>R recoding in mammalian gene *GRIA2* and I>V recoding in cephalopod gene *Kv2*), then the functions of the pre-edited or post-edited protein isoform should highly rely on the protein sequence which has already been fixed for a long period. In other words, if the Q of Q>R site is changed to another AA, then the protein might be malfunctioned, and there will be no need for RNA editing at all. Thus, DNA mutations changing the existing protein sequence are hardly tolerated and should be eliminated during evolution, leading to a depletion of ‘conserved editing with non-conserved recoding’ in current editomes. This prediction also aligns well with our intuition and a few known examples that conserved recoding tends to be functionally important.

In this study, we aim to (1) systematically identify and characterize conserved editing with non-conserved recoding in *Drosophila* editomes, emphasizing this less-noticed group of editing sites; (2) make an initial attempt to test whether DNA mutations leading to non-conserved recoding (that is, occurring in codons with conserved recoding events) are deleterious. We obtain a handful of such non-conserved recoding events and surprisingly find no signals of suppression (and instead, even observe positive selection) on these events at both inter-species and intra-species levels; (3) functional annotation found that the representative recoding sites were located in the domain regions of the proteins, potentially affecting their structures and functions. Our study suggests that conserved editing events with non-conserved recoding types might be functional as well and that we should not automatically reckon that only conserved recoding is functional based on the limited cases of experimental observations. Our study provides novel aspects in considering the adaptive evolution of RNA editing sites and proposed that the repertoire of functional recoding sites for future validation could be expanded.

## Materials and methods

### Phylogeny of Drosophila genus

We collected the reference genomes of 28 *Drosophila* species (**Supplementary Table S1**). According to the established phylogeny of *Drosophila* genus provided by FlyBase (https://flybase.org/), there are 18 available species ‘between’ *D. melanogaster* and *D. pseudoobscura*. We only utilized the topology of the tree, since our results did not rely on the branch length.

### Sequence alignment

For the edited coding genes in *D. melanogaster*, we selected the transcript with the longest CDS of each gene. We translate the CDS into protein and aligned their protein sequences those of other species with blastp [27]. Default parameters were used. The hit with the lowest E value was regarded as the orthologous genes in each species. Then, the orthologous sequences were aligned with mafft [28] with default parameters. CDSs were aligned according to the protein alignment. Since the edited genes in *Drosophila* generally have a high conservation level, the search for orthologs and the sequence alignment should be highly reliable and less sensitive to software, parameters, or cut-offs. The alignment of each codon/AA position was manually extracted from the sequence alignment file.

### Transcriptome mapping and variant visualization

BWA version 0.7.17 was used to map the RNA-Seq reads to the reference CDS sequence of the target species [29,30]. Default parameters were used. The sequence coverage and alignment at target region were visualized with IGV.

### Annotation of unedited adenosines

We split the reference genome of *Drosophila melanogaster* into single bases. In gene region, we extracted the adenosines. If the gene is located in the positive strand of the reference genome, then we should extract A in the reference genome sequence; if the gene is located in the negative strand of the reference genome, then we should extract T in the reference genome sequence. Presume A-to-I RNA editing occurs, then A in the positive strand genes should be replaced with G, and T in the negative strand genes should be replaced with C. Then, software SnpEff [31] was used to annotate the change caused by A-to-G. Nonsynonymous and synonymous changes were counted.

### Annotation of SNPs

The SNPs of *D. melanogaster* from DGRP project were also annotated by SnpEff [31]. In coding region, the software will tell us which codon this SNP is located, and thus we could infer the codon change and AA change based on this information. The nucleotide position on CDS and AA position on protein were also provided for each CDS SNP, and this enables us to match the SNPs with the genome-wide unedited adenosines, consequently determining which SNPs are located in conserved codons with nonsynonymous adenosines.

### Annotation, folding, and visualization of protein domains and structures

We used InterProScan v5 to annotate the domain regions of the protein sequences [32]. The resulting diagrams of protein domains were visualized using TBtools v1.108 [33], a biosequence structure illustrator. The protein secondary structure was visualized using PSIPRED program [34]. AlphaFold was performed by running the AlphaFold2 notebook on Google Collaboratory cloud computing facilities with default parameters. The Google Colab is accessible online at https://colab.research.google.com/github/phenix-project/Colabs/blob/main/alphafold2/AlphaFold2.ipynb. The resulting models were displayed with the PyMOL molecular graphics system [35]. Since the program allows a maximum length of 1000 AAs but both target proteins (Shab and CG16974) exceed this limitation, we therefore only folded the domains where the recoding sites were located. We folded the ion transport domain of Shab and the LRR (leucine-rich repeat) domain of CG16974.

The mass spectrum (MS) data were retrieved from a previous literature [36] and downloaded from ProteomeXchange (http://www.proteomexchange.org/) under accession number PXD009590. The peptides were searched against the reference protein sequences of Shab and CG16974 (FBtr0080489). Since the mismatch between the reference protein sequence and the post-edited peptide might preclude the detection of edited peptides, both the pre-editing and post-edited protein isoforms were used as reference sequences. Software MaxQuant v2.4.7.0 [37] were used with default parameters. The sequences and positions of identified peptides were recorded to profile the CDS-wide coverage and examine the existence of post-edited peptides. Note that the original literature did not report the detection of the two recoded peptides of our interest. This could be due to the different lists of editing sites used to modify the reference protein sequence. The post-edited peptide cannot be automatically identified if one did not provide the post-edited protein sequence as the reference. This is essentially different from the detection of A-to-I editing events from the RNA-Seq data where the mismatches between RNA reads and reference sequence can be directly seen from the alignment [38–41]. Another problem in peptide identification is the limitation of enzymatic cleavage site and the peptide length. The protein sequence can only be cleaved at K (Lys) or R (Arg). As our results showed, the predicted length of the peptide containing the editing site exceeded the maximum length of peptide identification, and thus the edited peptide cannot be captured.

### Statistical tests

Statistical tests were performed in R studio (R version 3.6.3). The graphical works were done in R environment.

## Results

### Direct searching of conserved editing with non-conserved recoding in Drosophila reveals signal of positive selection

To identify conserved editing with non-conserved recoding according to the strict definition, we retrieved the known lists of editing sites across three *Drosophila* species from our previous study: *D. melanogaster*, *D. simulans*, and *D. pseudoobscura*. Based on the 2114 high-confidence RNA editing sites in *D. melanogaster*, 996 editing sites are conserved between *D. melanogaster* and *D. simulans* (divergence time = 5.4 Mya), and 451 editing sites are conserved between *D. melanogaster* and *D. pseudoobscura* (divergence time = 55 Mya).

**Figure 2.**
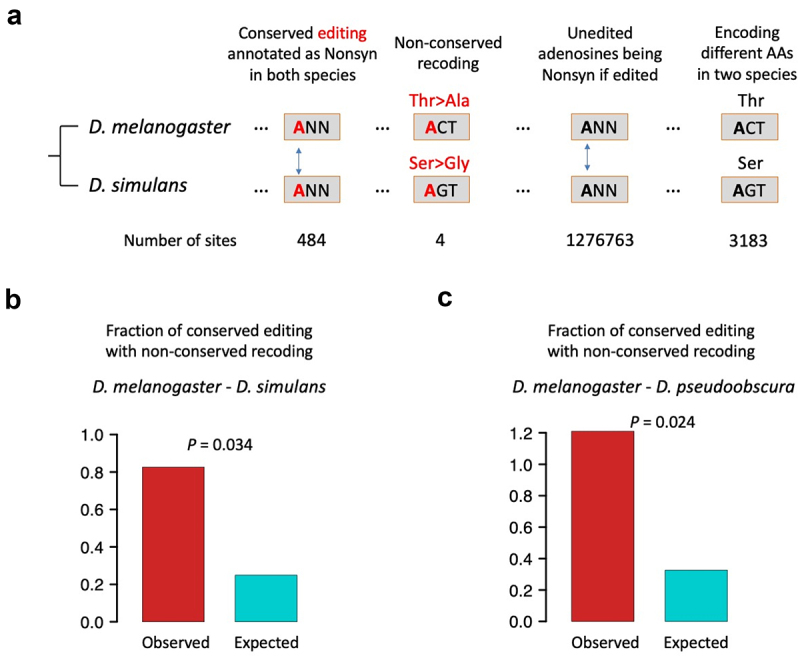
Observed and expected occurrences of conserved editing with non-conserved recoding between D. melanogaster and another sibling species. (a) Diagram illustrating how we look for the appearance of conserved editing with non-conserved recoding. The particular codons are just an example. (b) D. melanogaster - D. simulans comparison. p value was obtained by one-sided Fisher’s exact test. (c) D. melanogaster - D. pseudoobscura comparison. p value was obtained by one-sided Fisher’s exact test.

We obtained 484 *D. melanogaster-D. simulans* conserved editing sites annotated as nonsynonymous in both species, and 480 of them showed the same types of codon changes caused by A-to-G alteration. In other words, we found four conserved editing sites with non-conserved recoding between *D. melanogaster* and *D. simulans* (Figure 2a). This low fraction (0.826%) seems to indicate that this case is unlikely to appear between closely related species, probably because mutations on edited codons have not occurred or have not been fixed yet. As a control, we examined the unedited adenosines in edited genes. Between *D. melanogaster* and *D. simulans*, there are 1,276,763 codons with a conserved but unedited adenosine which will be annotated as nonsynonymous if A is mutated to G. Among them, 3183 codons encode different AAs in the two species. That is to say, if the 1,276,763 adenosines were edited, then the expected ratio of conserved editing with non-conserved recoding would be 3183/1276763 = 0.249% (Figure 2a). This expected fraction is significantly lower than the observed 0.826% (Figure 2b), suggesting the overrepresentation or positive selection on conserved editing with non-conserved recoding.

With similar strategy, we looked at the 329 *D. melanogaster-D. pseudoobscura* conserved editing sites annotated as nonsynonymous in both species and found that four sites are located in codons encoding different AAs and thus the editing sites recode differently (Figure 2c). The expected ratio of such non-conserved recoding is 4105/1257866 = 0.326%, which is significantly lower than the observed one 4/329 = 1.22% (Figure 2c). This again suggests that the situation of conserved editing with non-conserved recoding, although with only a handful of observations, is not suppressed and even has a tendency of overrepresentation and positive selection during evolution.

### Inference and confirmation of conserved editing with non-conserved recoding in the phylogeny

**Figure 3.**
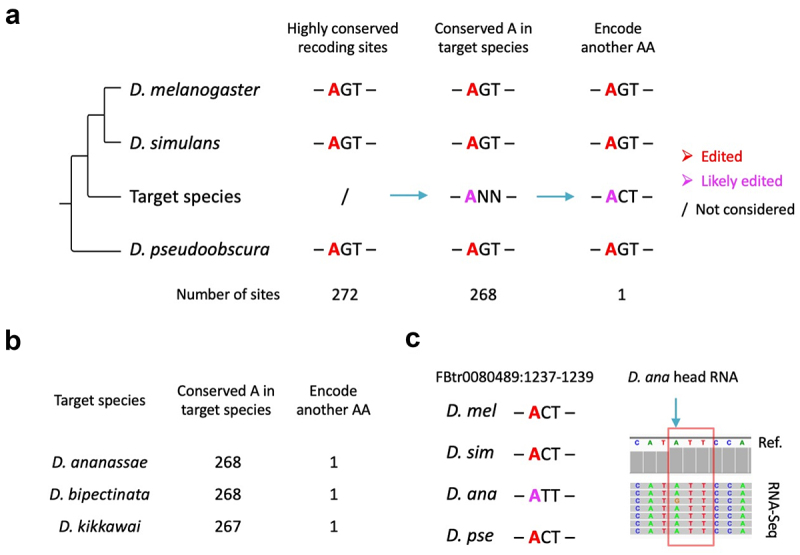
Inference of conserved editing with non-conserved recoding based on phylogeny. (a) Schematic diagram on how to search for the desired cases in a target species. (b) Summary of all cases found in 18 Drosophila species between D. melanogaster and D. pseudoobscura. (c) Visualization of particular cases where the codon in target species has changed but the orthologous adenosine is not edited. Edited adenosines in our three species are colored in red.

Due to the limited number of species with detailed RNA editomes, we only found a few cases of conserved editing with non-conserved recoding between particular *Drosophila* species. Nevertheless, there are other approaches to infer such potential cases from the phylogeny of *Drosophila* genus. We obtained 272 conserved editing sites that are annotated as nonsynonymous editing in all three species *D. melanogaster*, *D. simulans*, and *D. pseudoobscura* (Figure 3a). Given the rarity of editing-loss events as suggested by our previous studies [42–44], it could be temporarily presumed (although waiting for validation) that these 272 sites are edited in all the 18 available species ‘between’ *D. melanogaster* and *D. pseudoobscura* if the orthologous site is adenosine in the target species (Figure 3a and refer to **Materials and Methods** to see how the 18 species were obtained). If any species exhibit a different AA at the same position in the alignment, then this case will be the example of conserved editing with non-conserved recoding (Figure 3a).

In total, among the 272 highly conserved editing sites, we found one case of different AA in the genomes of *D. ananassae*, *D. bipectinata*, and *D. kikkawai*, respectively (Figure 3b). To test whether these diverged codons are really edited in target species, we searched for public head/brain transcriptomes within *Drosophila* genus and found RNA-Seq of heads of *D. ananassae* (**Materials and Methods**). In *D. melanogaster*, *D. simulans*, and *D. pseudoobscura*, RNA editing leads to a Thr>Ala (ACT>GCT) recoding, while in *D. ananassae*, the recoding will be Ile>Val (ATT>GTT) if editing really exists. We retrieved the orthologous gene corresponding to *D. melanogaster* gene *CG16974* (transcript ID FBtr0080489) and mapped the transcriptome reads to the *D. ananassae* mRNA sequence. RNA editing is indeed detected in *D. ananassae* (Figure 3c), suggesting that this methodology based on large-scale alignment is also capable of finding the conserved editing sites with non-conserved recoding. Nevertheless, compared to the direct searching approach in the above section, the methodology here is restricted to the limited numbers of species with available head/brain transcriptomes, or otherwise the putative cases cannot be verified.

### DNA polymorphism is overrepresented in codons with conserved recoding

As described above, the inter-species conserved editing sites with non-conserved recoding unlikely came from the independent gain of editing events at already diverged DNA sequences, and instead, it is likely gained by later DNA mutations in one of the two species with ancient conserved recoding event. If such DNA mutations are deleterious due to their damage to the conserved codon, then they should be suppressed at the beginning of emergence. At intra-species level, the single nucleotide polymorphisms (SNPs) in population provide a landscape for the selection on recent mutations. We downloaded the SNPs of global population of *Drosophila melanogaster* from the *Drosophila melanogaster* genetic reference panel (DGRP) [26]. We totally obtained 176,899 nonsynonymous and 342,617 synonymous SNPs. We questioned whether nonsynonymous SNPs are depleted in codons with conserved recoding?

**Figure 4.**
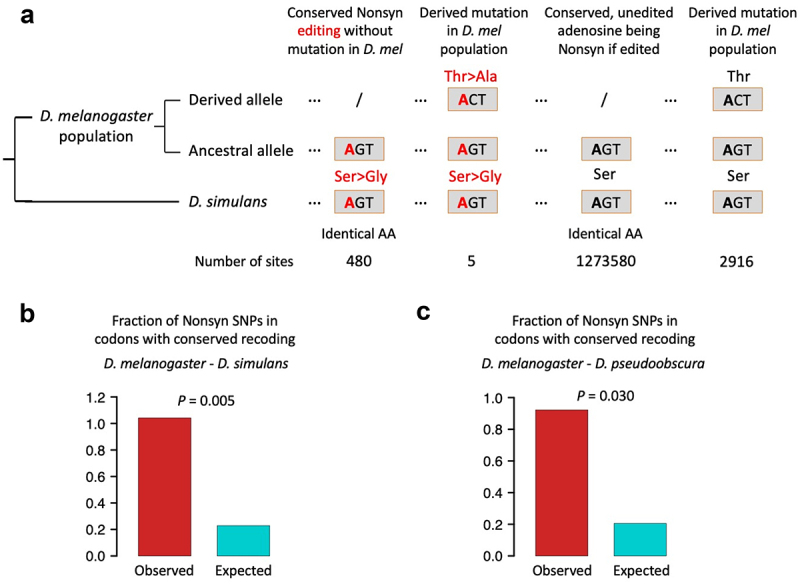
Fraction of nonsynonymous SNPs in codons with conserved recoding events. (a) The schematic diagram showing how we calculated the observed and expected fractions of SNPs. (b) Observed and expected fractions of SNPs. The D. melanogaster - D. simulans conserved recoding sites were used. p value was calculated by one-sided Fisher’s exact test. (c) Observed and expected fractions of SNPs. The D. melanogaster-D. pseudoobscura conserved recoding sites were used. p value was calculated by one-sided Fisher’s exact test.

Among the 484–4 = 480 conserved recoding sites between *D. melanogaster* and *D. simulans*, 5 recoding sites appear to have a nonsynonymous SNP located at the other nucleotides of the same codon, and thus the fraction is 5/480 = 1.042% (Figure 4a). Then, we use the unedited adenosines as negative control (Figure 4a). Considering that edited adenosines are generally more conserved than unedited ones, we only focus on edited genes to control for conservation level. In those genes, there are 1,273,580 codons: (1) contain unedited adenosine; (2) will be nonsynonymous if A-to-G occurs; and (3) the codons are conserved between *D. melanogaster* and *D. simulans* (Figure 4a). Among them, 2916 codons have a nonsynonymous SNP at the non-A nucleotides, and thus the expected fraction should be 2916/1273580 = 0.229% (Figure 4a). Again, it turns out that the observed fraction of nonsynonymous SNPs is significantly higher than the expected value (Figure 4b). Contrary to our intuition that this kind of nonsynonymous SNPs should be deleterious and depleted in codons with conserved recoding, we observed signal of positive selection on these mutations, suggesting a potential benefit of conserved editing with non-conserved recoding.

Similarly, among the 329–4 = 325 conserved recoding sites between *D. melanogaster* and *D. pseudoobscura*, 3 recoding sites appear to have a nonsynonymous SNP located at the other nucleotides of the same codon, and thus the fraction is 3/325 = 0.923% (Figure 4c). In contrast, within edited genes, 1,253,761 codons contain unedited adenosine and are conserved between *D. melanogaster* and *D. pseudoobscura*, among which 2573 codons have a nonsynonymous SNP at the non-A nucleotides, so the expected fraction is 2573/1253761 = 0.205% (Figure 4c). Again, we observed a significant preference on such mutations compared to neutral expectation.

In the comparison between *D. melanogaster* and either sibling species, the nonsynonymous SNPs that abolish conserved recoding (and lead to conserved editing but non-conserved recoding) are not suppressed and even significantly favoured by natural selection. The only explanation is that the consequence of which is not deleterious and might be beneficial instead. Therefore, given the data in hand, we draw a conclusion that conserved editing with non-conserved recoding is a special type of editing site that has comparable benefit with the widely studied conserved recoding sites. In the future functional studies, this set of sites should not be ignored. Instead, it will be an interesting issue to investigate how the distinct recoding in different species provides adaptation to the organisms.

### Inference of the functional impact of recoding sites

The functional impact of recoding sites should be reflected at the proteomic level. For the conserved editing sites with non-conserved recoding stressed in this study, we try to reveal their impact on protein function from the following aspects.

**Figure 5.**
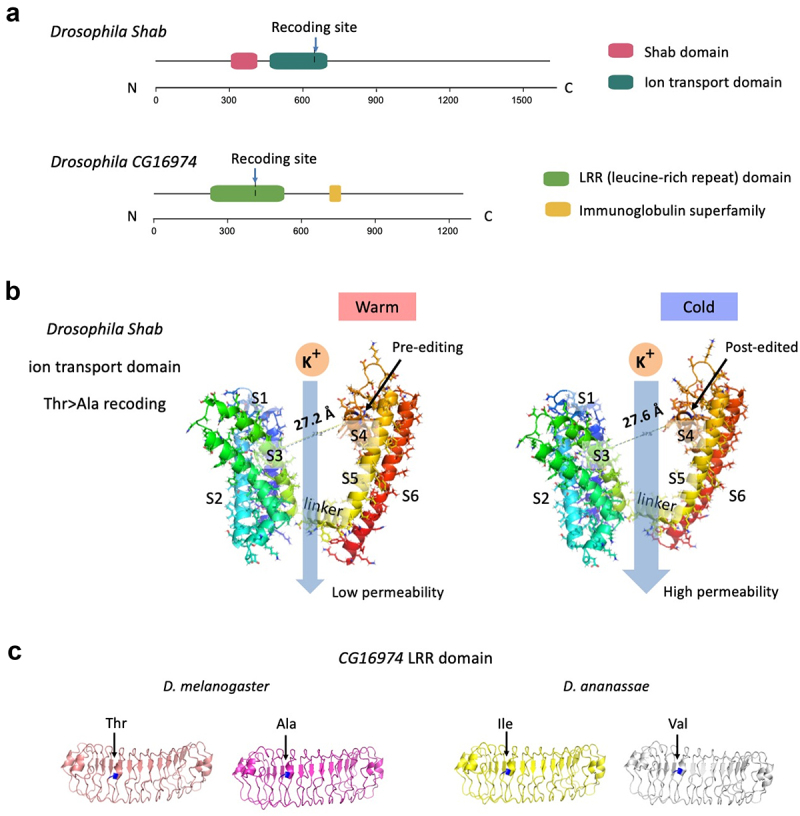
Protein domain and structure and the potential effect of RNA recoding events. (a) Protein domains of Drosophila Shab and CG16974. The locations of the recoding sites were labelled. (b) Domain structures of Drosophila Shab. The pre-editing and post-edited isoforms were displayed separately. Hydrogen bonds were shown. Editing site was pinpointed in the plot. The subunits (including S1~S6 and a linker region) were labeled according to reference [45]. (c) Domain structures of Drosophila CG16974. The pre-editing and post-edited structures were displayed separately. The recoded AAs were labelled in blue.

First, we interrogated whether the recoding events are located in the protein domains. The AA changes in domains are intuitively more impactful than the changes in linker regions. We used InterProScan to annotate the domains of *Shab* and *CG16974* (FBtr0080489) (**Materials and Methods**). We found that in both genes the recoding sites were located in domains: *Shab* encodes a potassium channel and the *Shab* recoding site was located in the ion transport domain; gene *CG16974* was less studied, but the recoding site was located in the LRR (leucine-rich repeat) domain (Figure 5a). These results suggest a potential effect of recoding events on the protein function.

Next, we used AlphaFold to construct the structures of the pre-editing and post-edited protein isoforms. In our previous study in *C. chinensis*, the Ser>Gly recoding in *Shab* did not seem to drastically change the protein structure, but since the assembly and annotation in *C. chinensis* was not as perfect as in model organisms, we reserve the possibility that the Shab protein function might be fine-tuned by this recoding event [24]. Here, we study the potential effect of Thr>Ala recoding in *D. melanogaster Shab* gene. The pre-editing and post-edited structures were adjusted to the same angle (Figure 5b). According to a recent comprehensive review [45], the ion transport domain of Shab consists of seven main helixes including S1–S6 and a linker. S1–S3 are the sensor domain, and S4–S6 are the pore-forming domain. The movement and conformational change of S4 helix directly controls the membrane potential and channel activity, where the distance between S4 and the sensor domain is a crucial determinant [45]. Interestingly, the *Drosophila* Thr>Ala recoding site is located in S4 (Figure 5b). We measured the distance between the recoded AA in S4 and its nearest AA in the sensor domain, representing the diameter of the pore. We discovered that the recoded Ala isoform had larger distance (27.6 Å) than the unedited Thr isoform (27.2 Å) (Figure 5b). This conformational change might affect the dynamics of the potassium channel where larger diameter enables higher ion permeability. More amazingly, similar to our previous finding in *C. chinensis* that this recoding level elevated under cold stress [24], in *Drosophila* the editing level at this site was also anti-correlated with temperature [43]. Thus, the recoded Shab protein with putatively higher ion permeability represents the cold-specific isoform (Figure 5b). These knowledges suggest that the insects might utilize recoding as a strategy to facilitate ion transportation and compensate the lower activity of Shab under cold temperature. Nevertheless, we acknowledge that a robust conclusion would only be made when experimental evidence was shown.

For the LRR domain of protein CG16974, the recoding events in both *D. melanogaster* and *D. ananassae* made discernable but not striking changes to the domain structures (Figure 5c), indicating a limited impact of RNA editing on protein function. Nevertheless, it should be noted that the functional change by a point mutation is not necessarily reflected by the structural changes, and we reserved the possibility that these conserved RNA editing sites with non-conserved recoding types might be an essential means to regulate the neuronal activity in different insects.

**Figure 6.**
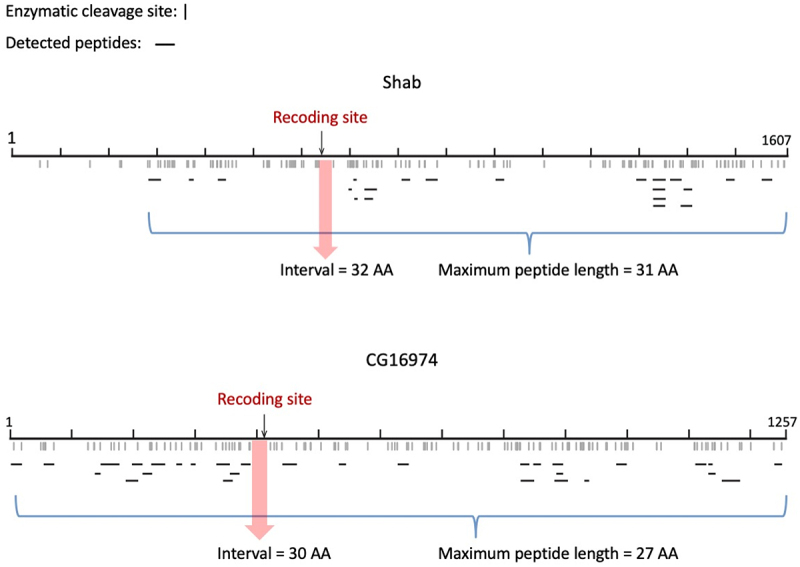
Identification of the post-edited peptides from the MS data. Shab and CG16974 were examined. The enzymatic cleavage sites (K or R) were labeled with “|”. All detected peptides were illustrated as “–” in the plot. The positions of recoding sites were highlighted. For the region containing the recoding site, the interval between two nearest cleavage sites exceeded the maximum length of peptide in the MS data and therefore the post-edited peptide could not be detected.

Then, given the potential functional impact of these recoding events, we tried to utilize the mass spectrum (MS) data of *D. melanogaster* heads to validate that the post-edited RNAs were indeed translated into proteins (**Materials and Methods**). We found two literatures that studied the effect of A-to-I RNA editing on *D. melanogaster* proteome [36,46] and one of which particularly generated head transcriptome [36]. However, the two original studies did not identify the two recoding sites of our interest [36,46]. We therefore searched the MS data against the pre-editing and post-edited protein sequences of Shab and CG16974 (**Materials and Methods**) and plotted the CDS-wide peptide coverage (Figure 6). An issue in peptide identification is the limitation of enzymatic cleavage site and the peptide length. The protein sequence can only be cleaved at K (Lys) or R (Arg). The enzymatic cleavage will produce a series of peptides of different lengths. However, the MS data usually identify peptides with lengths between 6 AAs and 30 AAs [37]. In Shab, we identified 22 peptides, and the maximum length was 27 AAs. We confirmed that all 22 peptides ended with K or R. However, the predicted length of the interval containing the Thr>Ala recoding site was 30 AAs, exceeding the maximum length of identified peptide (Figure 6). Thus, the recoded peptide in Shab could not be captured. Similarly, in CG16974, the cleaved interval containing the recoding site was 32 AAs, which exceeded the maximum length of the 30 detected peptides (Figure 6).

The inability to capture the post-edited peptides was only due to technical limitations and did not deny the translation of recoded CDS to protein. Moreover, the fact that the recoding sites located in domains of Shab and CG16974 caused slight conformational change of the protein structure further indicated the power of RNA editing to alter the protein functions and facilitate adaptation.

## Discussion

### Reconsidering the definition of conserved recoding sites

In this study, we proposed that the conserved editing sites with non-conserved recoding are likely to be functional and positively selected during evolution. These findings expanded the candidates of functional RNA editing sites for future validation. This special group of sites might automatically be ignored by researchers because they actually belong to an intermediate state of the traditionally defined conserved recoding site and non-conserved editing site. These conserved editing sites with non-conserved recoding were conceptually complicated and were not prioritized in functional studies.

In the continuous searching of potential functional RNA editing sites, the reason for focusing on conserved recoding site is apparent. The ‘molecular error theory’ [47] assumes that many of the observed molecular processes like RNA editing are random errors produced by cellular machineries and that only the few functional sites or events could be selectively maintained. In contrast, the ‘adaptive hypothesis’ believes that the RNA editing mechanism is a well-designed strategy used for increasing proteomic diversity [25] or restoring ancestral allele [48,49] and that the most essential sites are highly conserved during evolution. The common point of different theories is that the conserved RNA editing sites are likely to be functional [50]. This notion is supported by afore-mentioned cases of indispensable recoding sites conserved across many species.

However, the highly conserved and functional recoding events leave us an impression that only the strictly conserved recoding type, like Q>R recoding in all tested mammals, is functional and adaptive. In this work, made the first step towards understanding this rare situation. By defining conserved editing with non-conserved recoding, we demonstrated their special characteristics and the signal of positive selection acting on these sites. In particular, their appearance was overrepresented compared to random expectation, suggesting that this special type of editing site has an advantage to let natural selection select for the corresponding mutations. Thus, we propose that the definition of conserved recoding site should be updated. Non-conserved recoding might be functional as well and should not be ignored in the experimental studies. Promisingly, the addition of this set of sites might retrieve some interesting cases that broaden our thoughts on the biological significance of RNA editing.

### Why is non-conserved recoding tolerated?

The next question is why non-conserved recoding could be tolerated? If conserved recoding is so essential that the pre-edited and post-edited protein isoforms have their distinct functions being fixed during evolution, then any changes in protein sequence should be deleterious.

We delve into the case of *Shab* gene where S>G recoding is seen in *D. melanogaster* and T>A recoding is found in *C. chinensis*. With the outgroup DNA sequence available, it can be inferred by parsimonious approach that the ancestral state of *D. melanogaster* (Diptera) and *C. chinensis* (Hemiptera) was likely an (AGT>GGT) Ser>Gly recoding at this position, and then in Diptera the AGT codon was mutated to ACT but the editing event was maintained, leading to a Thr>Ala recoding. To explain how *Drosophila* could live with the Thr>Ala recoding after the mutation in its codon context, we propose that the ~360 Mya divergence between Hemiptera and Diptera enables a drastic change in the physiology, morphology, and behaviour of the insects. A conspicuous discrepancy is that *D. melanogaster* and *C. chinensis* are exposed to completely different habitats where *C. chinensis* is more tolerant to cold stress and harsh environments than *Drosophila*. Since gene *Shab* encodes a potassium channel and the recoding site locates in the domain region, fruitfly might have adjusted the function of Shab by AA substitution to adapt to a more moderate environment (e.g. adjusting the kinetics of the ion channel). However, even within the relatively moderate environment, there are still fluctuations of surrounding factors so that the flexibility of RNA editing is still needed.

It seems that when two species or two clades have diverged, living in completely different habitats and facing different extent of selection pressure, then the ancient conserved recoding site in one of the two species might simply ‘wait for’ the occurrence of a mutation to change the codon and AA. Most of such innovations were unsuccessful, but there were always a few adaptive mutations that were subsequently fixed in a clade. Compared to the efforts in understanding the function of conserved recoding sites, this case of conserved editing with non-conserved recoding is even more interesting due to the species-specific recoding type and its potential connection to the phenotype and adaptation of that species.

## Conclusions

Conserved editing with non-conserved recoding might be functional as well. One should not automatically reckon that only conserved recoding is essential based on limited cases of experimental studies. The candidates of functional recoding sites for future validation could be expanded.

## Abbreviations

AA: amino acid.
A-to-I: adenosine-to-inosine.
ADAR: adenosine deaminase acting on RNA.
CDS: coding sequence.
DGRP: *Drosophila melanogaster* genetic reference panel.
LRR: leucine-rich repeat.
MS: mass spectrum.
SNP: single nucleotide polymorphism.

## Supplementary Material

TableS1.docx

## Data Availability

The genome accession IDs of all *Drosophila* species used in this study were downloaded from NCBI (https://www.ncbi.nlm.nih.gov/), and the accession links were listed in Supplementary Table S1. The lists of high-confidence A-to-I RNA editing sites in brains of *Drosophila melanogaster*, *Drosophila simulans*, and *Drosophila pseudoobscura* were retrieved from our previous study. The SNP data were downloaded from the *Drosophila melanogaster* genetic reference panel (DGRP) [26]. The head transcriptomes of *D. ananassae* were downloaded from NCBI with accession IDs SRR7243208, SRR7243209, and SRR7243210. The mass spectrum (MS) data were retrieved from a previous literature [36] and downloaded from ProteomeXchange (http://www.proteomexchange.org/) under accession number PXD009590.

## Data availability

The genome accession IDs of all *Drosophila* species used in this study were downloaded from NCBI (https://www.ncbi.nlm.nih.gov/), and the accession links were listed in **Supplementary Table S1**. The lists of high-confidence A-to-I RNA editing sites in brains of *Drosophila melanogaster*, *Drosophila simulans*, and *Drosophila pseudoobscura* were retrieved from our previous study. The SNP data were downloaded from the *Drosophila melanogaster* genetic reference panel (DGRP) [26]. The head transcriptomes of *D. ananassae* were downloaded from NCBI with accession IDs SRR7243208, SRR7243209, and SRR7243210.
