# Supplementary material for: Conserved A-to-I RNA editing with non-conserved recoding expands the candidates of functional editing sites
Source: Fly (Austin). 2024 Jun 18;18(1):2367359. doi: 10.1080/19336934.2024.2367359 (PMC11188811; doi:10.1080/19336934.2024.2367359)
Supplement: TableS1.docx [file KFLY_A_2367359_SM6039.docx]

| **Order** | **Species** | **Data Source** | **Accession Number** | **Link** |
| --- | --- | --- | --- | --- |
| Diptera | *Drosophila ananassae* | NCBI | GCF_017639315.1 | https://ftp.ncbi.nlm.nih.gov/genomes/all/GCF/017/639/315/GCF_017639315.1_ASM1763931v2 |
| Diptera | *Drosophila biarmipes* | NCBI | GCF_018148935.1 | https://ftp.ncbi.nlm.nih.gov/genomes/all/GCF/018/148/935/GCF_018148935.1_ASM1814893v1 |
| Diptera | *Drosophila bipectinata* | NCBI | GCF_018153845.1 | https://ftp.ncbi.nlm.nih.gov/genomes/all/GCF/018/153/845/GCF_018153845.1_ASM1815384v1 |
| Diptera | *Drosophila elegans* | NCBI | GCF_018152505.1 | https://ftp.ncbi.nlm.nih.gov/genomes/all/GCF/018/152/505/GCF_018152505.1_ASM1815250v1 |
| Diptera | *Drosophila erecta* | NCBI | GCF_003286155.1 | https://ftp.ncbi.nlm.nih.gov/genomes/all/GCF/003/286/155/GCF_003286155.1_DereRS2 |
| Diptera | *Drosophila eugracilis* | NCBI | GCF_018153835.1 | https://ftp.ncbi.nlm.nih.gov/genomes/all/GCF/018/153/835/GCF_018153835.1_ASM1815383v1 |
| Diptera | *Drosophila grimshawi* | NCBI | GCF_018153295.1 | https://ftp.ncbi.nlm.nih.gov/genomes/all/GCF/018/153/295/GCF_018153295.1_ASM1815329v1 |
| Diptera | *Drosophila hydei* | NCBI | GCF_003285905.1 | https://ftp.ncbi.nlm.nih.gov/genomes/all/GCF/003/285/905/GCF_003285905.1_DhydRS2 |
| Diptera | *Drosophila kikkawai* | NCBI | GCF_018152535.1 | https://ftp.ncbi.nlm.nih.gov/genomes/all/GCF/018/152/535/GCF_018152535.1_ASM1815253v1 |
| Diptera | *Drosophila mauritiana* | NCBI | GCF_004382145.1 | https://ftp.ncbi.nlm.nih.gov/genomes/all/GCF/004/382/145/GCF_004382145.1_ASM438214v1 |
| Diptera | *Drosophila melanogaster* | NCBI | GCF_000001215.4 | https://ftp.ncbi.nlm.nih.gov/genomes/all/GCF/000/001/215/GCF_000001215.4_Release_6_plus_ISO1_MT |
| Diptera | *Drosophila miranda* | NCBI | GCF_003369915.1 | https://ftp.ncbi.nlm.nih.gov/genomes/all/GCF/003/369/915/GCF_003369915.1_D.miranda_PacBio2.1 |
| Diptera | *Drosophila mojavensis* | NCBI | GCF_018153725.1 | https://ftp.ncbi.nlm.nih.gov/genomes/all/GCF/018/153/725/GCF_018153725.1_ASM1815372v1 |
| Diptera | *Drosophila novamexicana* | NCBI | GCF_003285875.2 | https://ftp.ncbi.nlm.nih.gov/genomes/all/GCF/003/285/875/GCF_003285875.2_DnovRS2.1 |
| Diptera | *Drosophila obscura* | NCBI | GCF_018151105.1 | https://ftp.ncbi.nlm.nih.gov/genomes/all/GCF/018/151/105/GCF_018151105.1_ASM1815110v1 |
| Diptera | *Drosophila persimilis* | NCBI | GCF_003286085.1 | https://ftp.ncbi.nlm.nih.gov/genomes/all/GCF/003/286/085/GCF_003286085.1_DperRS2 |
| Diptera | *Drosophila rhopaloa* | NCBI | GCF_018152115.1 | https://ftp.ncbi.nlm.nih.gov/genomes/all/GCF/018/152/115/GCF_018152115.1_ASM1815211v1 |
| Diptera | *Drosophila sechellia* | NCBI | GCF_004382195.1 | https://ftp.ncbi.nlm.nih.gov/genomes/all/GCF/004/382/195/GCF_004382195.1_ASM438219v1 |
| Diptera | *Drosophila serrata* | NCBI | GCF_002093755.1 | https://ftp.ncbi.nlm.nih.gov/genomes/all/GCF/002/093/755/GCF_002093755.1_Dser1.0 |
| Diptera | *Drosophila simulans* | NCBI | GCF_016746395.2 | https://ftp.ncbi.nlm.nih.gov/genomes/all/GCF/016/746/395/GCF_016746395.2_Prin_Dsim_3.1 |
| Diptera | *Drosophila subobscura* | NCBI | GCF_008121235.1 | https://ftp.ncbi.nlm.nih.gov/genomes/all/GCF/008/121/235/GCF_008121235.1_UCBerk_Dsub_1.0 |
| Diptera | *Drosophila subpulchrella* | NCBI | GCF_014743375.2 | https://ftp.ncbi.nlm.nih.gov/genomes/all/GCF/014/743/375/GCF_014743375.2_RU_Dsub_v1.1 |
| Diptera | *Drosophila suzukii* | NCBI | GCF_013340165.1 | https://ftp.ncbi.nlm.nih.gov/genomes/all/GCF/013/340/165/GCF_013340165.1_LBDM_Dsuz_2.1.pri |
| Diptera | *Drosophila takahashii* | NCBI | GCF_018152695.1 | https://ftp.ncbi.nlm.nih.gov/genomes/all/GCF/018/152/695/GCF_018152695.1_ASM1815269v1 |
| Diptera | *Drosophila teissieri* | NCBI | GCF_016746235.2 | https://ftp.ncbi.nlm.nih.gov/genomes/all/GCF/016/746/235/GCF_016746235.2_Prin_Dtei_1.1 |
| Diptera | *Drosophila virilis* | NCBI | GCF_003285735.1 | https://ftp.ncbi.nlm.nih.gov/genomes/all/GCF/003/285/735/GCF_003285735.1_DvirRS2 |
| Diptera | *Drosophila willistoni* | NCBI | GCF_000005925.1 | https://ftp.ncbi.nlm.nih.gov/genomes/all/GCF/000/005/925/GCF_000005925.1_dwil_caf1 |
| Diptera | *Drosophila yakuba* | NCBI | GCF_016746365.2 | https://ftp.ncbi.nlm.nih.gov/genomes/all/GCF/016/746/365/GCF_016746365.2_Prin_Dyak_Tai18E2_2.1 |
